# MBFTFuse: A Triple-Path Adversarial Network Based on Modality Balancing and Feature-Tracing Compensation for Infrared and Visible Image Fusion

**DOI:** 10.3390/s26072109

**Published:** 2026-03-28

**Authors:** Mingxi Chen, Bingting Zha, Rui Yang, Yuran Tan, Shaojie Ma, Zhen Zheng

**Affiliations:** School of Mechanical Engineering, Nanjing University of Science and Technology, Nanjing 210018, China; chenmx@njust.edu.cn (M.C.); zhabingting@njust.edu.cn (B.Z.); ruiy@njust.edu.cn (R.Y.); tanyr@njust.edu.cn (Y.T.); shaojiem@njust.edu.cn (S.M.)

**Keywords:** image fusion, modality bias, feature tracing, adversarial learning

## Abstract

**Highlights:**

**What are the main findings?**

- This work proposes a triple-path GAN-based framework to fuse infrared and visible images, leveraging fused features for guidance and independent features for enhancement.
- The method outperforms state-of-the-art models on three datasets in both metrics and visual quality, excelling in downstream object detection.

**What are the implications of the main findings?**

- This study provides new insights into dual-modality fusion by demonstrating the effectiveness of a triple-path architecture for balancing feature guidance and enhancement.
- This study provides a novel research perspective for robust vision solutions in complex illumination conditions.

**Abstract:**

Infrared and visible image fusion aims to integrate complementary information from heterogeneous images captured by different optical sensors based on distinct imaging principles; however, existing methods often exhibit modality bias, leading to weakened targets or the loss of crucial texture details. To address this, we propose MBFTFuse, an adversarial fusion network based on modality balancing and feature tracing, which consists of a triple-path generator and dual discriminators. The architecture employs a generator with a triple-path structure: a central modality-balancing path for deep feature fusion and dual edge feature-tracing paths for modality-specific enhancement. Specifically, a multi-cognitive modality-balancing module is introduced to achieve feature weight equilibrium, while a Feature-Tracing Attention Module self-enhances single-modality features to compensate for information loss in the fusion results. Furthermore, a pixel loss based on intensity histograms is designed to optimize inter-modal balance at the pixel level. Comparative experiments against nine state-of-the-art methods across three public datasets demonstrate that MBFTFuse effectively highlights infrared targets while preserving intricate visible textures. The superior performance of this method in both quantitative metrics and downstream object detection tasks contributes to extending the boundaries of sensor-driven computer vision technologies.

## 1. Introduction

Compared with the single-modality image, the multi-modality fusion image not only contains more information due to the amount of data, but also has richer expressiveness in the visual perception level [1]. The visible image aligns with the human visual system, offering abundant texture details, yet it is susceptible to variations in illumination and occlusions. In contrast, the infrared image is generated based on thermal radiation signals from objects, providing prominent target saliency but lacking detailed descriptions. The proposal of infrared and visible image fusion (IVIF) technology aims to integrate the strengths of both modalities by combining their complementary information into a single image, thereby achieving a more comprehensive and robust representation of the scene [2]. However, the IVIF task often suffers from modality bias due to the inherent contrast dominance of infrared modality, causing the fused output to disproportionately favor infrared characteristics while attenuating visible texture representation, thereby creating an imbalance in cross-modality feature integration [3,4]. A critical challenge in current IVIF research lies in achieving balanced fusion of inter-modality information while effectively extracting complementary features from both modalities.

Prior to the widespread application of deep learning in computer vision tasks, image fusion primarily relied on handcrafted fusion rules implemented in either the spatial or transform domain [5]. Such methods typically required customized fusion strategies for different image combinations and reconstructed the fused image through inverse transformations. Traditional approaches—including sparse representation [6,7], multi-scale transforms [8,9] subspace methods [10,11], variational models [12] and hybrid models [13], aimed to achieve information fusion by modeling overlapping feature regions of different modalities in latent spaces and designing appropriate mathematical frameworks. However, these conventional methods heavily depended on manual rules and mathematical modeling. Consequently, they struggled to handle complex real-world scenarios, restricting their effectiveness in broader applications.

Convolutional Neural Networks (CNNs) have demonstrated outstanding performance in image fusion tasks due to their powerful feature extraction capabilities [14]. Representative methods, such as fusion networks based on encoder–decoder architectures [15,16], establish dynamic correlations between forward and backward features through residual connections [17]. However, they still exhibit limitations in global information modeling and cross-modality complementarity exploitation. Recent transformer-based fusion architectures [18,19] have achieved improved performance. Nevertheless, their exponentially increased parameter counts introduce training difficulties and excessive computational costs. To enhance fusion efficiency, some end-to-end methods [20,21] attempt to extract complementary features across modalities while reducing model complexity. However, they fail to fundamentally address the imbalanced modality weighting issue.

Meanwhile, Generative Adversarial Network (GAN)-based fusion methods [22] formulate IVIF as an adversarial process between generation and discrimination, progressively enhancing the realism of fused images through the min–max optimization of generator and discriminator. From the early single discriminator method [23], to subsequent double discriminator networks [24,25,26], and further to attention-enhanced variants [27], these methods have achieved remarkable progress in improving fusion quality. However, the existing GAN-based methods primarily focus on feature enhancement and complementarity modeling, lacking effective regulation of inter-modality feature weights.

To address the aforementioned challenges, this paper proposes a novel infrared and visible image fusion network based on modality balancing and feature-tracing compensation, termed MBFTFuse. The method constructs an adversarial framework with a triple-path generator and dual discriminators. The generator adopts an encoder–decoder structure, which is composed of a center modality-balancing path with deep cascading of infrared and visible features and dual edge feature-tracing compensation paths for individual modalities. The center path achieves modality balance by guiding single-modality features using the fused representation, while the dual-edge paths perform self-enhancement of single-modality features through the interaction modeling of both global and local feature semantics. The integration of all three paths ultimately enhances modality equilibrium and visual quality in the fused image.

**Figure 1:**
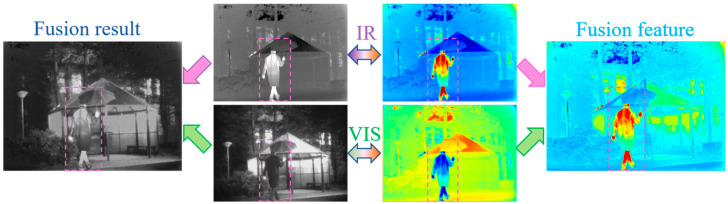
Feature visualization of MBFTFuse fusion process.

To intuitively demonstrate the fusion performance of MBFTFuse, we visualize the features of infrared and visible images from the fusion process in the TNO dataset (*Kaptein_1654*), as shown in Figure 1. The results indicate that the proposed method achieves both an accurate depiction of infrared targets and excellent visual perception of texture details in visible-light images.

The main contributions of this paper are summarized as follows:

- We propose a novel end-to-end adversarial fusion network, MBFTFuse, for infrared and visible image fusion. The generator employs a triple-path architecture with center modality balancing and dual edge feature-tracing, enabling multi-level feature extraction and effective cross-modality integration.
- We design two key modules to enhance fusion performance: the multi-cognitive modality-balancing module (MCMB) to adaptively regulate inter-modal feature weights in the center path, mitigating modality bias and the Feature-Tracing Attention Module (FTA) to reinforce single-modality features and enable more effective feature compensation. In addition, we have designed pixel loss based on intensity histograms, further optimizing the balance between modalities at the pixel level.
- Extensive experiments on three public datasets, namely TNO [28], MSRS [29], and RoadScene [30], including comparisons with nine state-of-the-art fusion methods and downstream object detection evaluation, demonstrate that MBFTFuse effectively highlights infrared targets while preserving visible textures. The proposed method achieves competitive performance in both qualitative and quantitative evaluations, and also shows strong practicality and robustness in the object detection task on the MSRS dataset, indicating its potential for multi-modal visual applications.

## 2. Related Work

This section systematically reviews GAN-based and CNN-based infrared and visible image fusion methods, with a critical analysis of their comparative advantages and inherent limitations.

### 2.1. GAN-Based Image Fusion Methods

Generative Adversarial Networks [22] are based on an adversarial game-theoretic framework between a generator and a discriminator: the generator aims to synthesize images that match the original data distribution in order to fool the discriminator, while the discriminator strives to distinguish real images from synthetic ones. Through this adversarial training process, the system ultimately reaches a Nash equilibrium.

GAN-based methods have demonstrated significant potential for image fusion tasks. The team of Ma et al. first proposed FusionGAN [31], constructing an end-to-end image fusion model, which they subsequently refined [23]. However, constrained by the discriminative capability of the single discriminator, the fused images still exhibited issues such as texture detail loss. To mitigate these limitations, DDcGAN [24] introduced a dual-discriminator structure, effectively enhancing the detail preservation capability of the fused images. Furthermore, GANMcC [25] reformulated fusion as a multi-distribution estimation problem. Meanwhile, Li et al. incorporated attention mechanisms into D2WGAN [26] to achieve image fusion. Subsequently, they proposed MgAN-Fuse [32] and AttentionFGAN [27], further extending the application of attention mechanisms in the field of image fusion.

Although these approaches have achieved certain success in highlighting key regional features, they insufficiently consider inter-modality complementarity during feature fusion.

### 2.2. CNN-Based Image Fusion Methods

Convolutional Neural Networks (CNNs) extract multi-level features through their layered architecture, significantly reducing the complexity of feature engineering compared to traditional fusion methods [14]. Li et al. [33] leveraged convolutional operations and residual connections [17] to extract image features, integrating them with Zero-phase Component Analysis to achieve image fusion. Subsequently, Li et al. [15] introduced an encoder–decoder architecture with dense residual connections, achieving notable performance. SwinFuse [18], built upon the transformer backbone [19], emphasized modeling long-distance dependencies. Huang et al. [34] proposed a dual-encoder architecture to fuse multi-level complementary information. More recently, transformer-based fusion methods have further strengthened long-range dependency modeling. SETFusion [35] introduces a semantic transformer to improve semantic-aware cross-modal representation learning, while DSKFuse [36] explores an efficient fusion framework based on dynamic sparse attention, KAN-based nonlinear mapping, and passive-active distillation learning. These studies reflect the recent trend toward semantic modeling and efficient cross-modal representation in IVIF. In addition, LIVFusion [37] uses wavelet transform and transformer architecture to solve the brightness and detail problems of fused images, but it is prone to overexposure.

Additionally, CrossFuse [20] introduced a cross-attention mechanism and adopted a two-stage training strategy to optimize the encoder and decoder separately, thereby improving fusion quality. MUFusion [38] utilized intermediate fusion results as supervisory signals to guide the fusion process; however, this training strategy is highly dependent on intermediate outputs and leads to unstable training. IRFS [39] incorporated multi-modal saliency object detection into the fusion process and enhanced cross-modality learning through multi-task collaboration, but this significantly increased the complexity and size of the network. VIF-Net [40] proposed an unsupervised infrared–visible image fusion framework that reduces the reliance on labeled data and improves fusion quality through end-to-end feature learning and tailored fusion constraints. Subsequently, U2Fusion [41] proposed an unsupervised end-to-end image fusion framework capable of adaptively preserving modality-specific features, but its limited control over features may lead to suboptimal fusion results.

## 3. Method

In this section, we first provide an overview of the overall architecture of the proposed MBFTFuse. Then, we focus on the design principles of the multi-cognitive modality-balancing module and the Feature-Tracing Attention Module, respectively. Finally, we explain the design of the loss function.

### 3.1. Modality Bias in Infrared–Visible Image Fusion

Infrared–visible image fusion aims to combine thermal saliency with rich textures. However, many deep learning methods suffer from modality bias: infrared information often dominates the fused representation, suppressing visible details [20]. This is especially evident in adversarial learning, where results emphasize thermal targets at the expense of fine structural textures [31].

In adversarial fusion frameworks, the generator *G* produces a fused image $I_{f}$ from the infrared image $I_{ir}$ and the visible image $I_{vis}$ as $I_{f}=G(I_{ir}+I_{vis})$. The training process follows the standard minimax objective as (1).

(1) $$\left\{\begin{array}{c} \min_{G}\max_{D}V(D,G)\\ V(D,G)=E_{x\sim p_{data}}[\log D(x)]+E_{I_{f}}[\log(1-D(I_{f}))] \end{array}\right.$$

From the distribution matching perspective, GAN optimization can be interpreted as minimizing the Jensen–Shannon divergence between the generated distribution $p_{g}$ and the target data distribution $p_{data}$ as (2).

(2) $$\underset{G}{\min}D_{JS}(p_{g}||p_{data})$$

However, in infrared–visible image fusion, the two modalities exhibit significantly different statistical characteristics. Infrared images usually produce stronger activation responses due to thermal radiation, while visible images mainly contribute high-frequency textures with relatively weaker amplitudes. As a result, the generator tends to minimize $D_{JS}(p_{g}||p_{ir})$ more easily than $D_{JS}(p_{g}||p_{vis})$, which causes the fused representation to gradually shift toward the infrared feature distribution.

Moreover, from the gradient propagation perspective, the optimization of the generator parameters can be expressed as (3). Since infrared features generally exhibit larger activation magnitudes than visible features, the corresponding gradients are typically stronger during backpropagation, resulting in $∥\nabla_{ir}∥>∥\nabla_{vis}∥$. Consequently, the optimization process tends to emphasize infrared structures while suppressing visible texture details, resulting in modality bias in the fused representation.

(3) $$\nabla_{\theta_{G}}L_{G}=\frac{\partial L}{\partial F_{ir}}\frac{\partial F_{ir}}{\partial \theta_{G}}+\frac{\partial L}{\partial F_{vis}}\frac{\partial F_{vis}}{\partial \theta_{G}}$$

To alleviate this imbalance, some studies introduce dual-discriminator architectures, where two discriminators $D_{ir}$ and $D_{vis}$ are used to enforce consistency with both infrared and visible modalities [24,27]. The adversarial objective can be expressed as (4).

(4) $$\left\{\begin{array}{c} \underset{G}{\min}\underset{D_{tr},D_{tis}}{\max}V(G,D_{ir},D_{vis})\\ \begin{array}{ll} V(G,D_{ir},D_{vis}) & =E_{I_{ir}}[\log D_{ir}(I_{ir})]+E_{I_{f}}[\log(1-D_{ir}(I_{f}))]\\ & +E_{I_{tis}}[\log D_{vis}(I_{vis})]+E_{I_{f}}[\log(1-D_{vis}(I_{f}))] \end{array} \end{array}\right.$$

This formulation encourages the fused image to simultaneously approximate both modality distributions. Nevertheless, due to the intrinsic statistical asymmetry between modalities, the generator may still favor structures that are easier to model during adversarial optimization. Other studies attempt to mitigate modality imbalance by introducing cross-attention mechanisms [20]. By dynamically reweighting feature responses from different modalities, cross-attention modules aim to preserve complementary information and enhance texture–target consistency in the fused representation. Despite these improvements, challenging scenarios may still lead to suboptimal fusion results.

To address the aforementioned modality bias issue, we propose MBFTFuse, which promotes effective interaction between fused features and modality-enhanced representations. Specifically, the generator integrates a single fusion branch with dual enhancement branches, while a dual-discriminator framework is employed to jointly regulate modality characteristics during adversarial learning. This design facilitates more balanced cross-modal feature representation and improves the quality of the fused images.

### 3.2. Overall Framework

**Figure 2:**
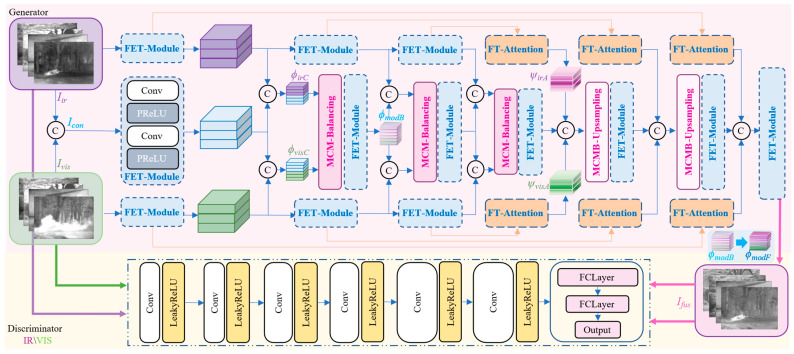
Architecture of MBFTFuse. The generator employs a triple-path structure, comprising a central modality-balancing path and dual edge feature-tracing paths. The discriminators adopt a dual-discriminator architecture with separate infrared and visible-light discriminators.

The overall architecture of the proposed network is illustrated in Figure 2. MBFTFuse adopts an encoder–decoder generator architecture and integrates dual discriminators for infrared and visible modalities to form an adversarial network.

Within the generator G, we construct a triple-path structure composed of a center modality-balancing path and dual edge feature-tracing paths. The input of the center path includes infrared image $I_{ir}\in {\mathbb{R}}^{H\times W\times 1}$, visible image $I_{vis}\in {\mathbb{R}}^{H\times W\times 3}$, and the concatenated image $I_{con}\in {\mathbb{R}}^{H\times W\times 2}$. The edge paths individually receive the original $I_{ir}$ and $I_{vis}$ images to enhance the self-attention and provide complementary features specific to each modality.

The dual discriminators share the same network architecture, designated as $D_{vis}$ and $D_{ir}$. Specifically, $D_{vis}$ takes the visible image $I_{vis}$ and the fused image $I_{fus}$ as input, guiding the generator to learn and preserve more texture and detail information from the visible modality. In contrast, $D_{ir}$ takes the infrared image $I_{ir}$ and the fused image $I_{fus}$ emphasizing the preservation and enhancement of salient infrared targets. During adversarial training, the generator iteratively refines its outputs under dual-discriminator supervision, ultimately synthesizing high-quality fused images that simultaneously preserve infrared target saliency and visible texture details.

### 3.3. Multi-Cognitive Modality-Balancing Module

**Figure 3:**
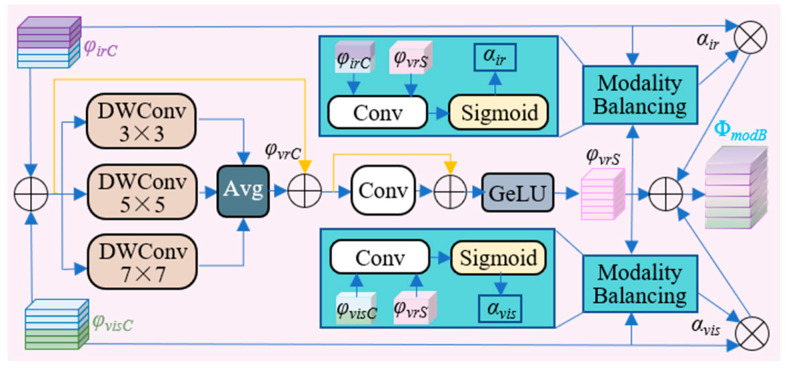
Architecture of MCMB. The fused features pass through a multi-cognitive convolutional filter bank, which then directs the generation of modality-specific weighting coefficients.

In IVIF tasks, the intensity saliency of infrared target regions leads them to receive higher response weights during feature extraction. Conversely, the detailed information in visible images becomes progressively suppressed through successive feature extraction stages [3,4]. This process can readily result in an imbalance in modality weighting [14]. To mitigate this modality imbalance, we propose the multi-cognitive modality-balancing module (MCMB). The architecture of the MCMB is illustrated in Figure 3.

We decompose visual information into multi-scale features, enabling the adapter to learn upstream features from multiple perspectives, and then further guides the allocation of weight coefficients for the different modalities. The inputs to MCMB are the concatenated feature maps from the center and edge paths, denoted as $\varphi_{visC}$ and $\varphi_{irC}$, respectively. These are summed to generate the initial fused feature map $\varphi_{vrC}$. During feature extraction, MCMB employs three parallel branches of DWConv [42] to decompose the visual information into multi-scale features. The channel-averaged features are then re-aggregated through a PointConv. Residual connections are introduced twice throughout this process to maintain feature consistency, while a GeLU activation enhances nonlinear representational capacity, yielding the guiding feature map $\varphi_{vrS}$. The overall process can be formalized as (5).

(5) $$\varphi_{vrS}=GeLU\left(\left(\varphi_{vrC}+Avg\left(\sum_{i=1}^{3}w_{dw}^{i}⊗\varphi_{vrC}\right)\right)\left(1+w_{pt}\right)\right)$$

where $w_{dw}^{i}\in {\mathbb{R}}^{C_{in}^{DW}\times K_{i}^{2}\times C_{out}^{DW}}\left(i\in 1,2,3\right)$ denotes the weights of the DWConvs with kernel sizes $K_{i}$, and $w_{pt}\in {\mathbb{R}}^{C_{in}^{PT}\times 1^{2}\times C_{out}^{PT}}$ represents the weights of the PointConv. ⊗ denotes the convolution operation.

Subsequently, the adapter leverages $\varphi_{vrS}$ as the guiding feature to derive the visible modality weight $\alpha_{vis}$ and infrared modality weight $\alpha_{ir}$, which are computed as (6) and (7), respectively.

(6) $$\alpha_{vis}=\sigma \left(w_{gp}⊗\left(Con\left[\varphi_{visC},\varphi_{vrS}\right]\right)\right)$$

(7) $$\alpha_{ir}=\sigma \left(w_{gp}⊗\left(Con\left[\varphi_{irC},\varphi_{vrS}\right]\right)\right)$$

where Con denotes the channel-wise concatenation operation, σ denotes the sigmoid function, and $w_{gp}\in {\mathbb{R}}^{C_{in}^{GP}\times 7^{2}\times C_{out}^{GP}}$ denotes the weights of the group convolution kernel.

Finally, the modality-balanced feature map $\Phi_{modB}$ is obtained via weighted summation, as in (8).

(8) $$\Phi_{modB}=\left(\alpha_{vis}\times \varphi_{visC}\right)+\left(\alpha_{ir}\times \varphi_{irC}\right)+\varphi_{vrS}$$

### 3.4. Feature-Tracing Attention Module

**Figure 4:**
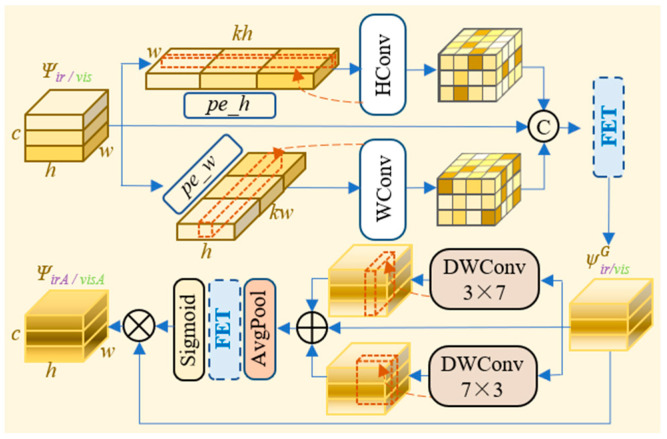
Architecture of the FTA. The FTA enhances single-modality features through self-attention reinforcement, by establishing semantic relationships between global and local features.

During the feature transmission process, the suppression or loss of important modality-specific information is often inevitable. Similar observations have also been reported in medical image restoration tasks, where explicit feature-guided designs are introduced to preserve diagnostically important structures during deep processing [43]. To improve the representational capability of source image features during fusion, we propose the Feature-Tracing Attention Module (FTA), inspired by prior attention mechanisms [44,45,46,47]. The architecture of the FTA is illustrated in Figure 4.

The FTA achieves feature tracing by performing global and local feature interacting on the original single-modality features. Specifically, the global feature interaction stage establishes long-range information flow and establishes a comprehensive semantic framework for the features. We first apply position embedding (*pe_h/w*) to the single-modality feature $\psi_{ir/vis}\in {\mathbb{R}}^{H\times W\times C}$ to establish implicit spatial relationships. Subsequently, the feature is partitioned along the channel dimension into *k* blocks. Each block is then reshaped along the H and W dimensions, yielding $\psi_{ir/vis}^{H}\in {\mathbb{R}}^{kH\times W\times \left(C/k\right)}$ and $\psi_{ir/vis}^{W}\in {\mathbb{R}}^{H\times kW\times \left(C/k\right)}$. The resulting feature maps are then processed separately using 3 × 3 convolutions to aggregate long-range information and then restored to original dimensions. These processed features are then concatenated with $\psi_{ir/vis}$, producing the global feature map $\psi_{ir/vis}^{G}\in {\mathbb{R}}^{H\times W\times C}$. This process can be formally expressed as (9).

(9) $$\psi_{ir/vis}^{G}=FET\left(Con\left[w_{H}⊗\psi_{ir/vis}^{H},w_{W}⊗\psi_{ir/vis}^{W},\psi_{ir/vis}\right]\right)$$

where *FET*(·) denotes the feature extraction and transformation operation, and $w_{H}\in {\mathbb{R}}^{C_{in}\times 3^{2}\times C_{out}}$ and $w_{W}\in {\mathbb{R}}^{C_{in}\times 3^{2}\times C_{out}}$ denote the weights applied along the H and W dimensions.

The local feature interaction stage focuses on learning local target features from infrared images and textural details from visible images. Specifically, we apply 3 × 7 and 7 × 3 convolutions to $\psi_{ir/vis}^{G}$ to extract directional local feature responses, respectively. The results are then summed with $\psi_{ir/vis}^{G}$. Subsequently, we achieve the single-modality attention feature maps $\psi_{irA/visA}\in {\mathbb{R}}^{H\times W\times C}$ after feature extraction and activation. This process can be formally expressed as (10) and (11), respectively.

(10) $$\psi_{visA}=\psi_{vis}^{G}\times \sigma \left(FET\left(AP\left(w_{dw}^{3*7}⊗\psi_{vis}^{G}+w_{dw}^{7*3}⊗\psi_{vis}^{G}+\psi_{vis}^{G}\right)\right)\right)$$

(11) $$\psi_{irA}=\psi_{ir}^{G}\times \sigma \left(FET\left(AP\left(w_{dw}^{3*7}⊗\psi_{ir}^{G}+w_{dw}^{7*3}⊗\psi_{ir}^{G}+\psi_{ir}^{G}\right)\right)\right)$$

where *AP*(·) denotes the global average pooling operation, $w_{dw}^{3\times 7}\in {\mathbb{R}}^{C_{in}\times 3\times 7\times C_{out}}$ and $w_{dw}^{7\times 3}\in {\mathbb{R}}^{C_{in}\times 7\times 3\times C_{out}}$ represent the weights of the two sets of local convolutional kernels, respectively.

### 3.5. Loss Function

In the MBFTFuse, we independently design loss functions for the generator and the dual discriminators. Beyond conventional reconstruction-oriented optimization, targeted feature-preserving constraints have also been shown to be beneficial in related image restoration tasks for preventing the suppression of important structures during deep processing [43]. Accordingly, the generator loss is composed of three components: the global loss $L_{global}$ for constraining feature information acquisition, the pixel loss $L_{pix}$ for balancing the pixel intensity distribution of fused images, and the adversarial loss $L_{adv}$ between the generator and discriminator. The expression is as (12).

(12) $$L_{G}=\mu_{1}L_{global}+\mu_{2}L_{pix}+\mu_{3}L_{adv}$$

where $\mu_{1}$, $\mu_{2}$ and $\mu_{3}$ denote the weighting coefficients for the loss terms.

Infrared features can be represented as single-channel intensity information, while the textural details of visible images can be characterized by gradients [31]. Using this information to construct the global loss $L_{global}$ as (13).

(13) $$L_{global}=\frac{1}{HW}\left({\left\|I_{fus}-I_{ir}\right\|}_{F}^{2}+\xi {\left\|\nabla I_{fus}-\nabla I_{vis}\right\|}_{F}^{2}\right)$$

where ${\left\|\cdot \right\|}_{F}$ denotes Frobenius norm. ∇ denotes the gradient operator, and ξ denotes the balancing factor.

Leveraging the characteristic that target pixels in infrared images stand out in intensity, we assign higher weights to the regions with high pixel contrast, thereby constructing a pixel weight matrix $M_{ir}\in {\mathbb{R}}^{H\times W}$ for the infrared image as (14).

(14) $$M_{ir}\left(x,y\right)=Norm\left(\sum_{p=1}^{256}P_{ir}\left(p\right)\left|I_{ir}\left(x,y\right)-p\right|\right)$$

where $P_{ir}\left(p\right)$ indicates the proportion of pixels with intensity value *p* in the infrared image intensity histogram. $I_{ir}\left(x,y\right)$ refers to the intensity value of the infrared image at spatial location $\left(x,y\right)$. Norm represents normalization operation.

The weight matrix for the visible image is constructed as $M_{vis}=1-M_{ir}$. The pixel loss $L_{pix}$ is expressed as (15).

(15) $$L_{pix}=\frac{1}{N}\sum_{n=1}^{N}\left({\left\|I_{fus}^{n}-I_{vis}^{n}⊙M_{vis}\right\|}_{1}+{\left\|I_{fus}^{n}-I_{ir}^{n}⊙M_{ir}\right\|}_{1}\right)$$

where N denotes the total number of images, ${\left\|\cdot \right\|}_{1}$ denotes the L1 norm and ⊙ denotes element-wise multiplication between the image and the weight matrix. $M_{vis/ir}\in {\mathbb{R}}^{H\times W}$ can be extended to ${\mathbb{R}}^{H\times W\times C}$ to match the image channels.

To further enhance the realism and modality balance of the fused image, we introduce an adversarial loss $L_{adv}$ based on dual discriminators. The formulation is defined as (16).

(16) $$L_{adv}=\frac{1}{2N}\sum_{n=1}^{N}\left({\left(D_{vis}\left(I_{fus}^{n}\right)-1\right)}^{2}+{\left(D_{ir}\left(I_{fus}^{n}\right)-1\right)}^{2}\right)$$

For the discriminators $D_{vis}$ and $D_{ir}$, the corresponding loss functions are defined as (17) and (18), respectively [25,26].

(17) $$L_{D_{vis}}=\frac{1}{N}\sum_{n=1}^{N}\left(D_{vis}\left(I_{fus}^{n},I_{vis}^{n}\right)+\lambda {\left({\left\|\nabla_{I_{vis}^{n}}D_{vis}\left(I_{vis}^{n}\right)\right\|}_{2}-1\right)}^{2}\right)$$

(18) $$L_{D_{ir}}=\frac{1}{N}\sum_{n=1}^{N}\left(D_{ir}\left(I_{fus}^{n},I_{ir}^{n}\right)+\lambda {\left({\left\|\nabla_{I_{ir}^{n}}D_{ir}\left(I_{ir}^{n}\right)\right\|}_{2}-1\right)}^{2}\right)$$

where λ is a penalty coefficient, ${\left\|\cdot \right\|}_{2}$ denotes the L2 norm.

## 4. Experiments and Analysis

This section first presents the model training setup and implementation details. Subsequently, we conduct both qualitative and quantitative comparisons to demonstrate the superiority of our network and then conduct ablation experiments. Finally, we assess the potential of the fused images in a downstream object detection task.

### 4.1. Training and Testing Details

(1) Dataset Settings: Experiments are conducted on three public datasets: TNO [28], MSRS [29] and RoadScene [30]. The model is trained exclusively on the TNO dataset and evaluated on all three datasets. For quantitative evaluation, we use image pairs from TNO (30 pairs), MSRS (50 pairs), and RoadScene (50 pairs). The object detection task employs labeled images from the MSRS dataset (80 pairs).

(2) Comparison Methods and Evaluation Metrics: To ensure a fair comparison, we evaluate both classical and recent state-of-the-art models using their respective source codes and pre-trained weights. The evaluation process strictly follows the original methodologies, keeping the testing environment and hyper-parameters identical across all baselines. The comparison methods are as follows: DenseFuse [15], FusionGAN [31], U2Fusion [41], PIAFusion [29], SeAFusion [47], IJMLC [34], IRFS [39], MUFusion [38], LIVFusion [37].

The following indicators are adopted for quantitative evaluation: Entropy (EN), Mutual Information (MI), Spatial Frequency (SF), Sum of Correlation Differences (SCD), Visual Information Fidelity (VIF), gradient-based fusion performance (Q^AB/F^), Structural Similarity Index Measure (SSIM). the specific meanings and computational details of which can be found in [48].

In the objective evaluation framework, EN and MI are employed to characterize the information abundance and the degree of source information inheritance, respectively. SF is utilized to measure the activity of microscopic details, while SCD and VIF assess the fusion quality from the perspectives of complementary feature correlation and human visual perception. Regarding the QAB/F and SSIM, their composite forms may obscure the relative contributions of the infrared and visible modalities. To provide a more detailed analysis, we further decompose them into modality-specific components, namely (Q^IR/F^, Q^VI/F^) and (SSIM^IR^, SSIM^VI^). This decomposition is intended to reveal the modality dependence of the fused image at both the detail level and the global structural level. Based on these quantities, we define the Detail Balance Score (DBS) and the Global Balance Score (GBS). The formulation is defined as (19) and (20), where $\varepsilon =1\times 10^{-6}$.

It should be noted that DBS and GBS are introduced as supplementary diagnostic indicators, rather than universal criteria for fusion quality. A higher DBS or GBS indicates lower dominance of one modality over the other under the same evaluation setting, but does not imply that an ideal fusion result should always approach an equal infrared–visible contribution ratio. Therefore, DBS and GBS should be interpreted jointly with Q_sum_, SSIM_sum_, and the corresponding scene characteristics. In this work, Q_sum_ and DBS are used together to analyze the inheritance and relative symmetry of detail information from the two modalities, while SSIM_sum_ and GBS are used together to examine the global structural consistency and the relative dominance of modality contributions.

(19) $$\left\{\begin{array}{c} Q_{sum}=Q^{IR/F}+Q^{VI/F}\\ DBS=1-\frac{\left|Q^{IR/F}-Q^{VI/F}\right|}{Q_{sum}+\varepsilon } \end{array}\right.$$

(20) $$\left\{\begin{array}{c} SSIM_{sum}=SSIM^{IR}+SSIM^{VI}\\ GBS=1-\frac{\left|SSIM^{IR}-SSIM^{VI}\right|}{SSIM_{sum}+\varepsilon } \end{array}\right.$$

(3) Training and Testing Details: A total of 18,828 pairs of 128 × 128 cropped images are randomly selected from 25 image pairs in the TNO dataset as training samples [28]. The batch size and epoch are set to 4 and 16, respectively, with the Adam optimizer used to update model parameters. During training, the generator is updated once after the discriminator iterates twice. The learning rate for the discriminator is set to 4 × 10^−4^ while the generator is set to 1 × 10^−4^. The coefficients in the loss function $\mu_{1}$, $\mu_{2}$, and $\mu_{3}$, are set to 1, 0.8, and 1, respectively [24,25,27,31]. The balance factor ξ in *L_global_* is set to 0.8 [31], and the penalty coefficient λ in the discriminator loss *L_Dvis_*/*L_Dir_* is set to 10 [25,26], respectively. Experiments are conducted on a platform with an AMD 5700X CPU, 32 GB memory, and an NVIDIA GeForce RTX 4090 GPU, using Python 3.9 and PyTorch 2.1.0 as the programming environment.

### 4.2. Comparison with SOTA Methods

**Figure 5:**
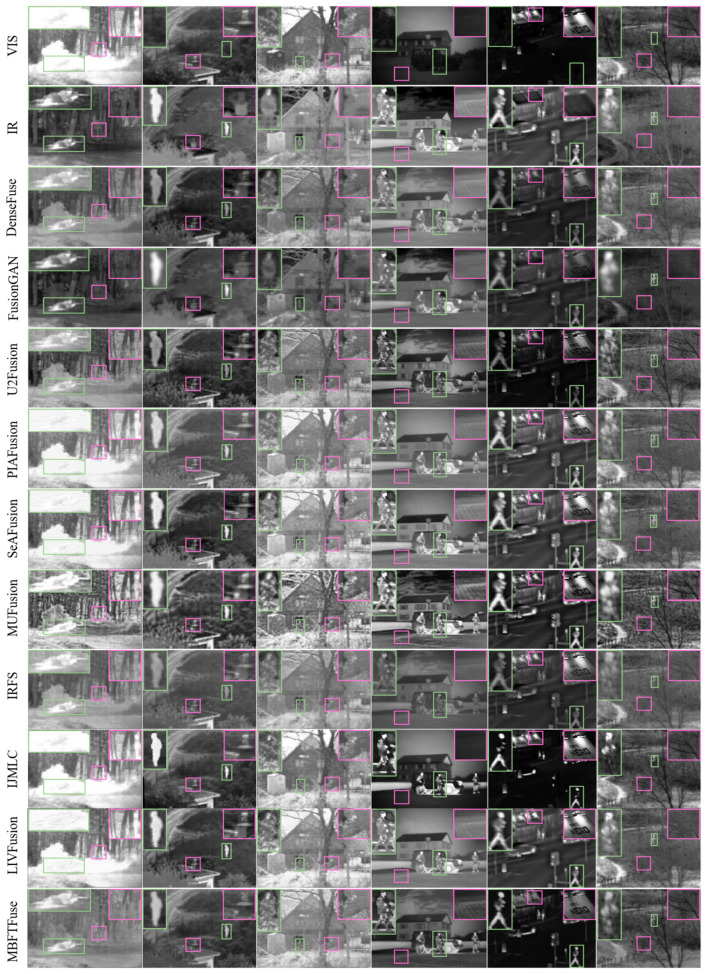
Qualitative comparison on the TNO dataset. Green bounding boxes highlight infrared target regions, while purple bounding boxes indicate textural detail regions. From left to right, the image pairs are: *soldier_behind_smoke_1*, *Nato_camp_sequence_1816i*, *man_in_doorway*, *soldiers_with_jeep*, *street*, *and sandpath*.

(1) Analysis of Fusion Results on the TNO Dataset: We selected six representative image pairs from the TNO dataset for visualization, as shown in Figure 5. Green boxes highlight infrared target regions, while purple boxes indicate detailed texture areas. (*soldier_behind_smoke_1*, *Nato_camp_sequence_1816i*, *man_in_doorway*, *soldiers_with_jeep*, *street, sandpath*).

Analysis of comparative results reveals the following: DenseFuse produces fused images with overall low saturation and a grayish appearance. FusionGAN exhibits noticeable feathering artifacts, resulting in blurred edges and loss of fine details. U2Fusion and IRFS fail to sufficiently highlight infrared features in small targets and occluded regions (e.g., *sandpath*). MUFusion delivers images with high sharpness and strong contrast but amplifies noise significantly. PIAFusion, SeAFusion, IJMLC, and LIVFusion achieve high contrast but suffer from modality imbalance in highlight regions. This manifests as confusion between infrared targets and bright scenes in visible images (e.g., *soldier_behind_smoke_1*) and lost texture details (e.g., pavement tiles and clouds in *soldiers_with_jeep*). In contrast, MBFTFuse accurately preserves thermal radiation characteristics from infrared images while retaining textures and details from visible images. Our method is relatively optimal in terms of visual representation.

The quantitative comparison is presented in Table 1. The proposed method achieves the best performance on the EN and MI metrics, indicating that the fused image retains the highest proportion of features during the fusion process. Concurrently, the optimal performance on the SF metric validates the superiority of our fused image in preserving textures and details. Furthermore, the best values on the VIF second-best performance on the SCD metrics signify that our fused images possess superior visual fidelity and exhibit less distortion.

**Figure 6:**
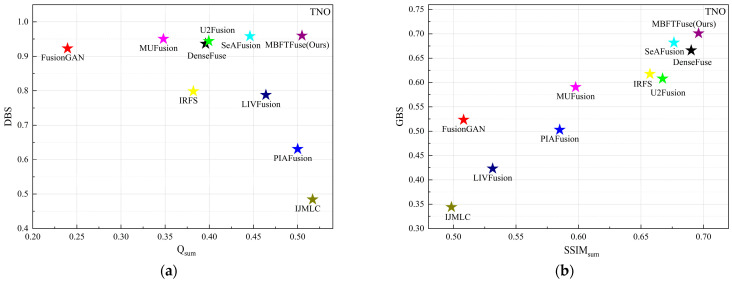
Quantitative evaluation of modality preservation and balance on the TNO dataset: (**a**) scatter plot of Q_sum_ vs. DBS for detail-level analysis; (**b**) scatter plot of SSIM_sum_ vs. GBS for global-level analysis.

Simultaneously, an integrated analysis of Table 1 (right half) and Figure 6 reveals that in the TNO dataset, the values of Q^VI/F^ generally surpass those of Q^IR/F^, whereas SSIM^IR^ is significantly greater than SSIM^VI^. This suggests that the edge details of the fused images primarily originate from the visible modality, while the holistic luminance, contrast, and structural information are more reliant on the infrared modality. Notably, the IJMLC method achieves the maximum Q^VI/F^ and exhibits the largest gap between SSIM^IR^ and SSIM^VI^ among all evaluated methods. In terms of holistic perception, its fused images feature intense contrast and sharp infrared target contours. However, its excessive reliance on the visible modality for texture extraction leads to the suppression of certain infrared targets or details by the visible background. For instance, the target in “*soldier_behind_smoke_1*” is obscured by smoke from the visible image, and infrared-derived details, such as the floor tile textures and clouds in “*soldiers_with_jeep*” are lost. Consequently, as shown in Figure 6, IJMLC is positioned in the bottom-right of (a) and the bottom-left of (b), respectively.

Meanwhile, methods located toward the right-hand side of the scatter plots achieve higher Q_sum_ or SSIM_sum_, while methods located toward the upper region exhibit lower dominance of a single modality under the same evaluation setting. The proposed method maintains competitive overall scores while also showing relatively balanced modality contributions, indicating that it can better coordinate local detail inheritance and global structural consistency.

**Figure 7:**
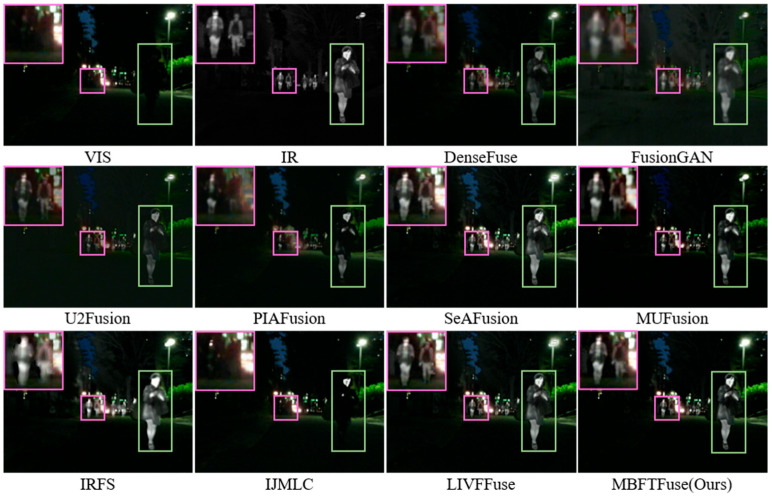
Qualitative comparison on the MSRS dataset (*00036N*). Green bounding boxes highlight large foreground targets, while purple bounding boxes indicate small distant targets.

(2) Analysis of Fusion Results on the MSRS Dataset: The MSRS dataset covers various lighting scenarios with multiple infrared targets, providing an ideal benchmark for evaluating fusion algorithms in detail fidelity. The qualitative example (*00036N*) is illustrated in Figure 7.

Compared to DenseFuse, FusionGAN, and IRFS, our proposed MBFTFuse achieves better contrast in large target regions and superior clarity and detail preservation in small target regions. Relative to LIVFusion, MUFusion, SeAFusion, and U2Fusion, our method demonstrates notable advantages in preserving texture and background details. Both PIAFusion and IJMLC show varying degrees of information loss in small-target areas.

**Figure 8:**
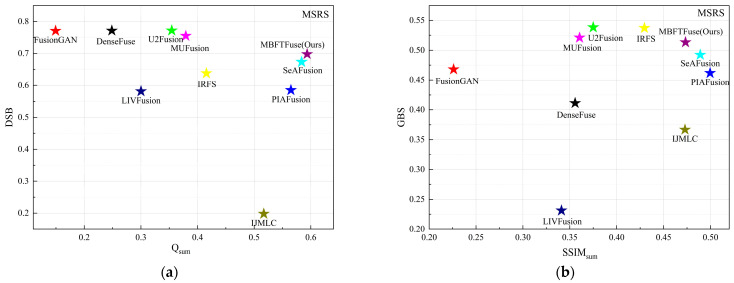
Quantitative evaluation of modality preservation and balance on the MSRS dataset: (**a**) scatter plot of Q_sum_ vs. DBS for detail-level analysis; (**b**) scatter plot of SSIM_sum_ vs. GBS for global-level analysis.

The quantitative results are summarized in Table 2. MBFTFuse achieves the best performance in four metrics: MI, SF, SCD, VIF, and ranks second in EN. As illustrated in Figure 8, although our method does not achieve the absolute optimal values across all individual metrics, it consistently occupies the top-right quadrant of the scatter plots in terms of comprehensive performance. This demonstrates that MBFTFuse is capable of maintaining superior fusion quality while simultaneously harmonizing holistic characteristics and fine-grained details from both modalities, even under the diverse illumination conditions of the MSRS dataset.

**Figure 9:**
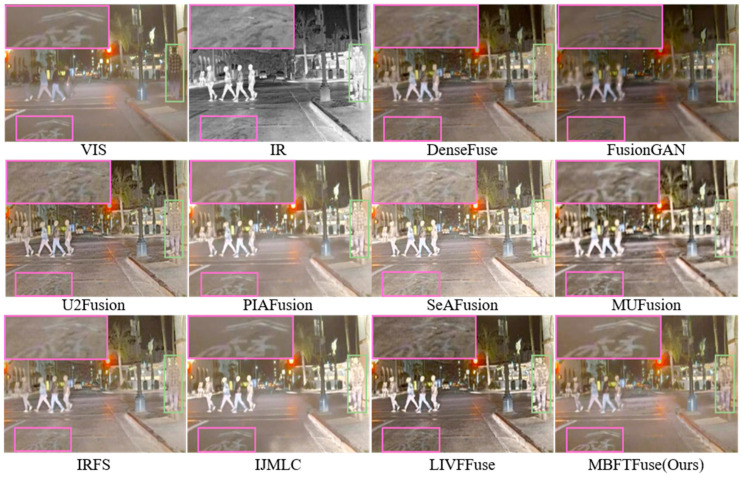
Qualitative comparison on the RoadScene dataset (*FLIR_08874*). Green bounding boxes highlight large foreground targets, while purple bounding boxes indicate small distant targets.

(3) Analysis of Fusion Results on the RoadScene Dataset: The RoadScene dataset contains daytime and nighttime street scenes, generally characterized by overexposure and low contrast. The qualitative example (*FLIR_08874*) is illustrated in Figure 9. The quantitative results are summarized in Table 3.

In terms of background detail reconstruction, only PIAFusion, IJMLC, and our proposed MBFTFuse are able to reconstruct the patterns clearly. These methods consistently exhibit higher Q^VI/F^ values, indicating a more extensive inheritance of background details from the visible-light images. Regarding pedestrian targets, although methods such as IJMLC and MUFusion provide intense contrast, they suffer from a significant loss of fine-grained details. Quantitatively, this is reflected in a larger disparity between SSIM^IR^ and SSIM^VI^ compared to other methods, which consequently prevents them from achieving a competitive GBS.

**Figure 10:**
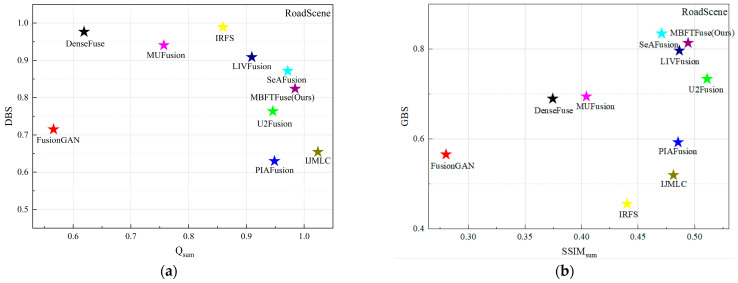
Quantitative evaluation of modality preservation and balance on the RoadScene dataset: (**a**) scatter plot of Q_sum_ vs. DBS for detail-level analysis; (**b**) scatter plot of SSIM_sum_ vs. GBS for global-level analysis.

In contrast, our proposed MBFTFuse not only maintains the saliency of the human target, but also better preserves textural details, such as the plaid pattern of the shirt, achieving a balanced fusion of visual clarity and information completeness. Simultaneously, in the scatter plots for the RoadScene dataset as Figure 10, compared with other methods, the proposed method achieves competitive overall scores and maintains relatively moderate modality dominance in both detail-level and global-level analyses.

### 4.3. Ablation Study

To verify the rationality of the algorithm design, two groups of ablation experiments were conducted on the TNO dataset (30 pairs of test images).

**Figure 11:**
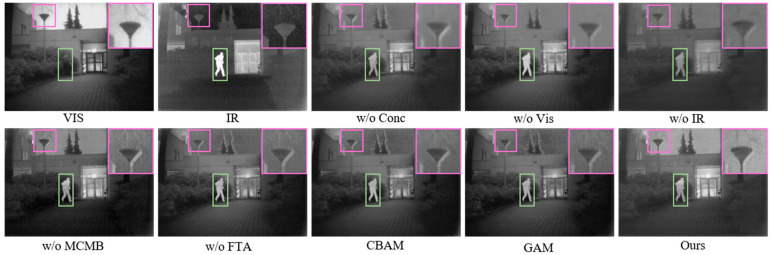
Qualitative comparison of network structure and module design on the TNO dataset. Green bounding boxes highlight large foreground targets, while purple bounding boxes indicate small distant targets.

(1) Effectiveness Analysis of Network Architecture and Module Design: Taking the TNO dataset (*Kaptein_1123*) a representative case, Figure 11 and Table 4 present the qualitative and the quantitative evaluation results, respectively.

In the network, we designed a center modality-balancing path and dual edge feature-tracing paths to mitigate modality imbalance and feature information attenuation. Removing the center path and edge paths, respectively, resulted in decreased image contrast, blurred structures, and missing details. Removing the MCMB caused imbalanced fusion relationships between the background and foreground. The FTA enhances image self-attention while aggregating single-modality features. We tested removing the FTA and replacing it with CBAM or GAM modules: though visual differences were insignificant, FTA outperformed others in evaluation metrics.

**Figure 12:**
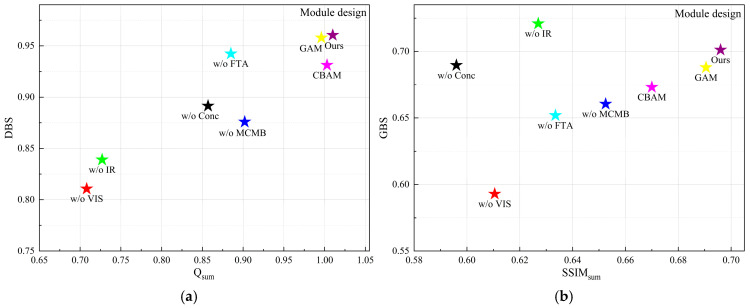
Quantitative evaluation of network structure and module design on the TNO dataset: (**a**) scatter plot of Q_sum_ vs. DBS for detail-level analysis; (**b**) scatter plot of SSIM_sum_ vs. GBS for global-level analysis.

Quantitative analysis reveals that ablating either the visible or infrared edge path leads to a decrease in the Q and SSIM values of the corresponding modality, resulting in the loss of source information. Meanwhile, removing the center path causes a simultaneous marginal decline in the metrics for both modalities. As illustrated in Figure 12, the absence of any single path degrades the overall performance. Furthermore, the incorporation of the MCMB boosts DBS and GBS by 9.59% and 6.05%, respectively, while Q_sum_ and SSIM_sum_ increase by 11.97% and 6.67%, representing a substantial enhancement in comprehensive performance. Notably, the FTA outperforms CBAM and GAM, yielding improvements of 1.91% and 7.51% in DBS and GBS, and 14.12% and 9.87% in Q_sum_ and SSIM_sum_, respectively. Meanwhile, our method achieved optimal values in EN, MI, SF, VIF and suboptimal values in SCD. In conclusion, these results thoroughly validate the effectiveness of the proposed triple-path architecture and the individual module designs.

**Figure 13:**
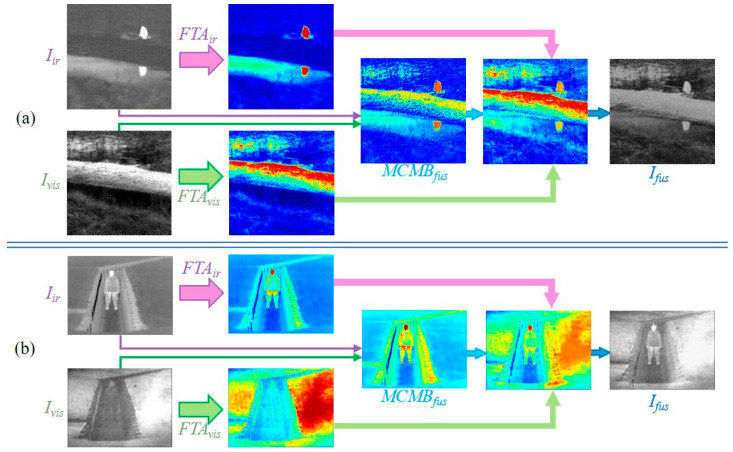
Visualization of feature compensation by the FTA and MCMB branches. (**a**) Visualization of “*bench*” image in the TNO dataset. (**b**) Visualization of “*soldier_in_trench_1*” image in the TNO dataset.

To further analyze the effect of the proposed FTA and MCMB, Figure 13 presents the output feature maps of the final-layer FTA branches, the corresponding MCMB feature, and their fused representation. The visualization shows that the MCMB branch is able to retain the major complementary information from the infrared and visible modalities, whereas its representation of fine-grained texture and structural details is still insufficient. In comparison, the FTA branches produce more discriminative responses in salient targets and local detail regions, especially around texture-rich and edge-aware structures. After integration with the MCMB feature, these responses effectively enhance the detail representation of the fused feature map. This demonstrates that the FTA can provide complementary modality-specific information for details that are not fully captured by the central fusion branch.

**Figure 14:**
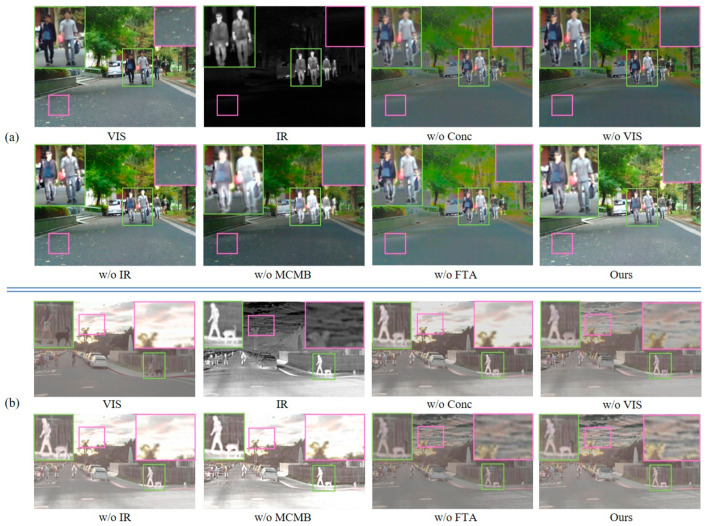
Qualitative comparison of network structure and module design on the MSRS and RoadScene datasets. Green bounding boxes highlight large foreground targets, while purple bounding boxes indicate small distant targets. (**a**) Visualization of “*01441D*” image in the MSRS dataset. (**b**) Visualization of “*FLIR_06422*” image in the RoadScene dataset.

To further verify the generalizability of the proposed core modules, we additionally conduct ablation experiments on the MSRS and RoadScene datasets. The corresponding quantitative results are shown in Table 5, and representative visual comparisons are provided in Figure 14, where Figure 14a shows MSRS image “*01441D*” and Figure 14b shows RoadScene image “*FLIR_06422*”. The results show that the proposed MCMB, FTA, and triple-path structure consistently improve fusion performance across different datasets, which further demonstrates that the effectiveness of these designs is not limited to the TNO dataset.

**Figure 15:**
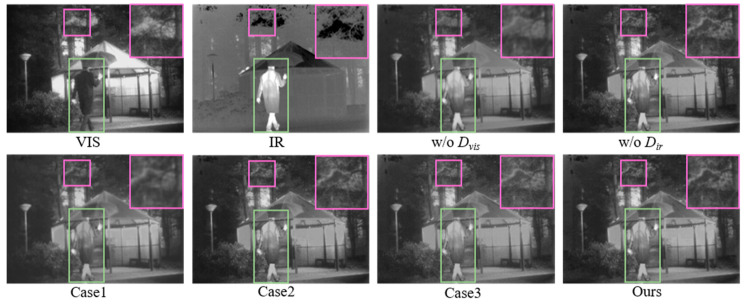
Qualitative comparison of the dual-discriminator and loss function design on the TNO dataset. Green bounding boxes highlight large foreground targets, while purple bounding boxes indicate small distant targets.

(2) Effectiveness Analysis of Dual-Discriminator and Loss Function Design: Taking the TNO dataset (*Kaptein_1654*) as a representative case, Figure 15, Table 6 and Table 7 present the qualitative and the quantitative evaluation results, respectively.

Visual analysis reveals that the dual-discriminator structure ensures superior rendering of infrared targets and background details in the fused image. The inclusion of $L_{global}$ guarantees holistic fusion quality; removing $L_{pix}$ results in detail loss, while removing $L_{adv}$ causes edge blurring and reduced contrast.

**Figure 16:**
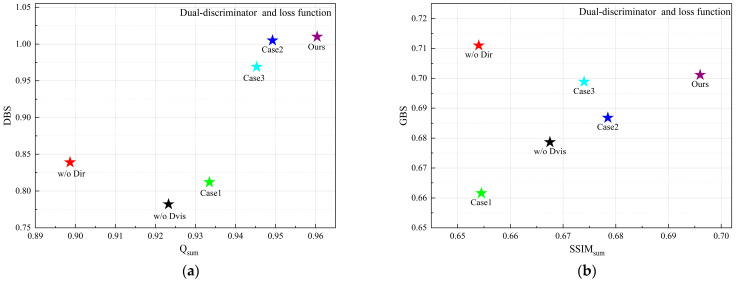
Quantitative evaluation of the dual-discriminator structure and loss function design on the TNO dataset: (**a**) scatter plot of Q_sum_ vs. DBS for detail-level analysis; (**b**) scatter plot of SSIM_sum_ vs. GBS for global-level analysis.

A quantitative analysis of Table 6 and Table 7, along with Figure 16, reveals that a single discriminator struggles to balance bi-modal features, often leading to performance degradation in the corresponding modality. The incorporation of $L_{pix}$ and $L_{adv}$ losses enhance the model’s capability in both local detail extraction and global feature representation. Compared to employing a single $L_{global}$ loss, our proposed method yields improvements of 2.89%, 5.89%, 24.38%, and 6.34% in DBS, GBS, Q_sum_, and SSIM_sum_, respectively, demonstrating a significant advancement in overall fusion quality.

To further analyze the influence of the proposed pixel loss weight, we conduct a parameter sensitivity analysis of *μ*_2_ on the TNO dataset with *μ*_1_ = 1 and *μ*_3_ = 1 fixed. The results are reported in Table 8. It can be seen that different values of *μ*_2_ lead to different trade-offs between pixel-level modality regulation and overall fusion performance. When *μ*_2_ is set to 0.8, the proposed method achieves relatively balanced performance across multiple metrics. Therefore, *μ*_2_ = 0.8 is selected in our experiments.

The MSRS test set contains samples captured under both daytime and nighttime illumination conditions. To evaluate the performance of $L_{pix}$ under different lighting conditions, we divide the test set into two subsets according to illumination: daytime (DT1/2, *01388D–01603D*, 50 image pairs) and nighttime (NT1/2, *00681N–00886N*, 50 image pairs), and conduct separate ablation experiments on each subset. The results are presented in Table 9.

Overall, the differences in performance between daytime and nighttime scenes are pronounced, which mainly stem from the distinct information distributions of the source images under different illumination conditions. In daytime scenes, visible images usually contain richer textures, edges, and structural details. As a result, the fused images generally achieve higher values on EN, MI, and SF, which reflect information content and spatial frequency, while Q^VI/F^ also indicates a stronger inheritance of visible-detail information. Under this condition, introducing $L_{pix}$ leads to improvements in EN, MI, SCD, Q^IR/F^, DBS, and SSIM^IR^, suggesting that this loss term can enhance the preservation of infrared-salient information at the pixel level and alleviate modality bias to a certain extent. However, since visible images in daytime already possess strong structural and textural advantages, the weights derived from the infrared intensity distribution may partially compress the representation space of high-frequency visible details, resulting in slight decreases in SF, VIF, Q^VI/F^, SSIM^VI^, and GBS. In contrast, under nighttime conditions, visible images suffer from low illumination, leading to evident degradation of details and contrast, whereas thermal targets in infrared images are typically more stable and salient. Consequently, the fused results rely more heavily on infrared information, which is reflected by higher values of Q^IR/F^ and SCD. Under such circumstances, the infrared weights derived by $L_{pix}$ provide a more reliable pixel-level prior. This not only further strengthens the preservation of thermally salient regions, but also promotes the retention of the remaining visible structural information under the joint constraints of the global loss and adversarial loss. Therefore, except for a slight decrease in DBS, the remaining metrics show relatively consistent improvements. These results indicate that $L_{pix}$ plays a positive role under both lighting conditions, while its benefit is clearly scene-dependent and becomes more significant in nighttime low-illumination environments than in daytime scenes.

### 4.4. Extended Experiment of Object Detection

**Figure 17:**
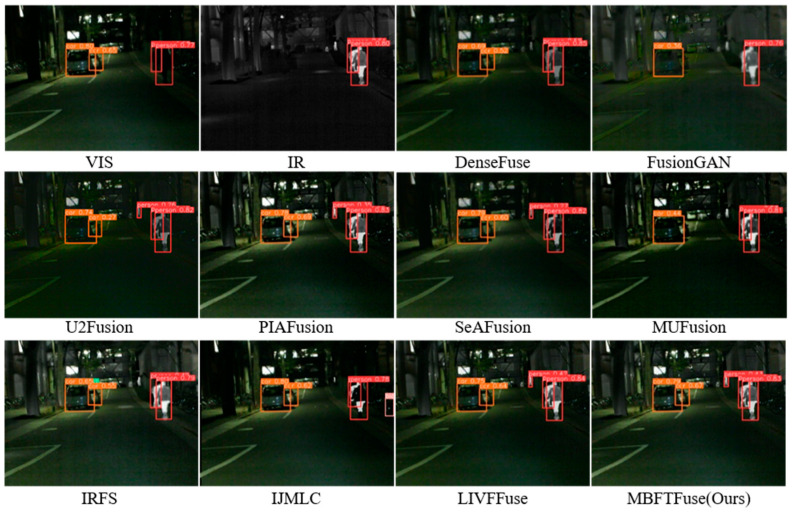
Object detection results on the MSRS dataset (*01049N*).

Infrared and visible image fusion should not only exhibit strong visual quality but also enhance downstream task performance. Based on the MSRS dataset, we employed the YOLOv8 [49] algorithm to evaluate the effectiveness of different fusion algorithms in an object detection task. Specifically, we use the original pre-trained YOLOv8n weights and directly apply the detector to the fused images without any additional fine-tuning or retraining on the fused-image domain [50]. The purpose of this experiment is to assess whether the fused images can improve object detectability under a ready-to-use detector setting, rather than to optimize the detector itself. The visualization results of object detection (*01049N*) are shown in Figure 17.

This scene, captured under low-light conditions, contains two “car” objects and three “person” objects. Only U2Fusion, PIAFusion, SeAFusion, LIVFusion and the proposed MBFTFuse methods successfully detect the small distant “person” target. Among them, MBFTFuse achieves the highest confidence score. In addition, we assess detection performance using three standard metrics: Precision, Recall, and mean Average Precision (mAP). The quantitative results are summarized in Table 10. Among all methods, MBFTFuse achieves the highest scores in both average Precision and mAP, and ranks second in average Recall. In summary, MBFTFuse demonstrates superior feature preservation and detection performance in multi-modal fusion scenarios, especially excelling in representing small-scale targets and fine-grained details.

### 4.5. Efficiency Analysis

To further evaluate the computational complexity of different methods, we set the input size to 640 × 480 × 1 following the image size setting of the MSRS dataset [29], and compute the parameter count (Params) and FLOPs using TorchSummary and Thop tools. The results are shown in Table 11. It can be observed that lightweight methods such as DenseFuse, PIAFusion and SeAFusion exhibit relatively low computational cost, while the proposed MBFTFuse introduces more parameters and FLOPs. This is mainly due to the triple-path generator and dual-discriminator design adopted to achieve stronger cross-modal interaction and more effective detail compensation. Although the proposed method is not the most lightweight, it achieves more competitive qualitative and quantitative fusion results. In future work, we will further investigate more lightweight architectures to reduce computational complexity while maintaining the fusion performance.

### 4.6. Thermal Crossover Challenges and Failure Case Analysis

**Figure 18:**
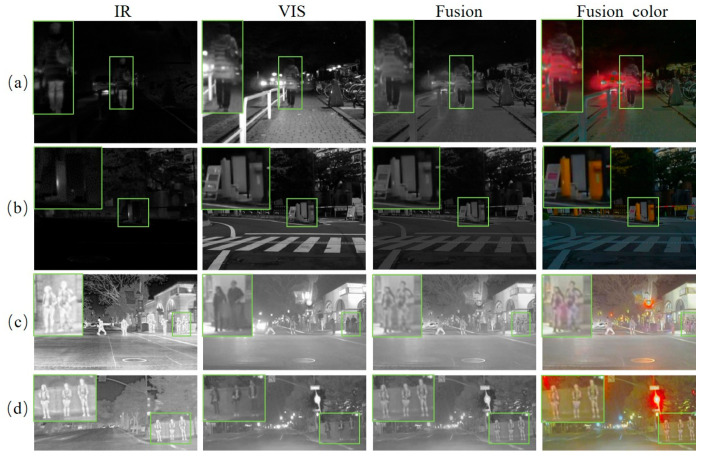
Thermal crossover challenges on the MSRS and RoadScene dataset. Green bounding boxes highlight large foreground targets, while purple bounding boxes indicate small distant targets. (**a**,**b**) Visualization of “*1178N*” and “*1574D*” images in the MSRS dataset. (**c**,**d**) Visualization of “*FLIR_06282*” and “*FLIR_video_02223*” images in the RoadScene dataset.

(1) Thermal Crossover Challenge Analysis: Thermal crossover refers to a challenging condition in which the thermal response of the background becomes close to that of the target, making the target less distinguishable in the infrared image. Such cases are particularly difficult for fusion methods, since the infrared modality can no longer provide a sufficiently strong saliency prior for target enhancement. Representative examples from the MSRS and RoadScene datasets are shown in Figure 18. where (a) and (b) correspond to MSRS “*01178N*” and “*01574D*”, respectively, and (c) and (d) correspond to RoadScene “*FLIR_06282*” and “*FLIR_video_02223*”, respectively.

It can be observed that the manifestation of thermal crossover differs across datasets. In the MSRS examples, both the targets and the surrounding background appear relatively dark in the infrared images, indicating that the thermal contrast is weak and the target response is largely suppressed. By contrast, in the RoadScene examples, both the targets and the background exhibit relatively high infrared responses, resulting in a bright overall thermal distribution in which target regions are easily mixed with high-response background areas. This discrepancy may be related to differences in sensor characteristics, imaging conditions, and scene-dependent thermal distributions across datasets.

Despite these challenges, the proposed method still produces reasonably good fusion results in both cases. In the MSRS examples, the target regions marked by the green boxes remain recognizable in the fused images even when the infrared saliency is weak. In the RoadScene examples, although the background also presents strong thermal responses, the proposed method is still able to preserve the target structures while maintaining the visible scene context. These results indicate that the proposed framework retains a certain degree of robustness under thermal crossover conditions, although such scenarios remain challenging for fully reliable target enhancement.

**Figure 19:**
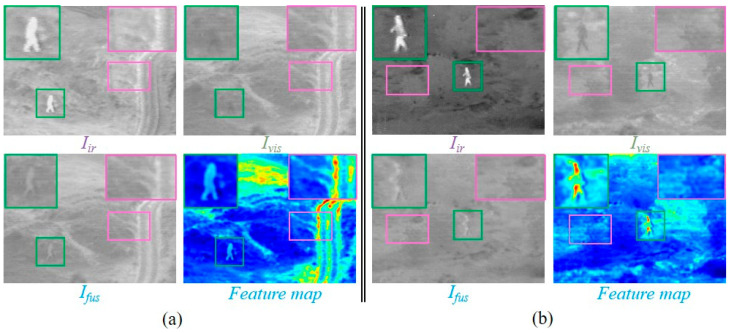
Failure case on the TNO dataset. Green bounding boxes highlight large foreground targets, while purple bounding boxes indicate small distant targets. (**a**) Visualization of “*Duine_sequence_7045*” image in the TNO dataset. (**b**) Visualization of “*Tree_sequence_4915*” image in the RoadScene dataset.

(2) Failure Case Analysis: Although the proposed method achieves satisfactory fusion performance in most scenarios, it still exhibits certain limitations in some challenging cases. To further analyze its shortcomings, we select two representative failure cases from the TNO dataset, as shown in Figure 19 (*Duine_sequence_7045* and *Tree_sequence_4915*). Specifically, in Figure 19a, corresponding to “*Duine_sequence_7045*”, the infrared image contains a pedestrian target with clear thermal saliency, while the visible image mainly provides weak ground texture and structural information. From the fused result, it can be observed that the small target marked by the green box is significantly weakened, whereas the texture details within the purple-boxed region are relatively well preserved. A similar phenomenon can also be observed in Figure 19b.

We further attempt to interpret this phenomenon from the perspective of the proposed model design. The MCMB in the central branch is designed to balance the dominant information from the two modalities, but weak small-region responses may still be overwhelmed during feature aggregation. Although the FTA branches are introduced to trace and compensate modality-specific details, their effectiveness still depends on whether these weak details can produce sufficiently discriminative activations in the original modality-specific features. For extremely weak targets or subtle textures, such responses remain limited and are therefore difficult to fully enhance in the final fused representation. In addition, since the proposed *L_pix_* is derived from the global infrared intensity distribution, its guidance may also be less sensitive to very small targets whose contribution to the global intensity histogram is limited.

## 5. Conclusions

To address the modality bias issue in IVIF tasks, we propose a novel fusion network, termed MBFTFuse. The network adopts a triple-path generator coupled with a dual-discriminator architecture. Within the center path, fused features are utilized to guide the generation of weighting coefficients for the individual modality features, thereby achieving modality balance. Concurrently, the dual edge paths perform feature self-enhancement on the single-modality inputs, enabling feature compensation during the fusion process. Finally, pixel loss was designed to further eliminate the imbalance between modalities at the pixel level.

Comprehensive evaluations were conducted across three publicly available datasets. Experimental results demonstrate that our proposed method consistently outperforms state-of-the-art approaches in both subjective visual quality and objective quantitative metrics. The high precision and robustness of MBFTFuse make it an excellent fusion solution for various multi-modal visual sensor tasks.

Limitations: Our method is mainly intended to address modality bias in infrared–visible image fusion. Nevertheless, in some challenging cases, such as scenes containing weak targets, subtle local textures, or complex background interference, the proposed framework may still show limited capability in preserving inconspicuous targets and recovering fine details. Moreover, the triple-path generator and dual-discriminator framework lead to relatively high model complexity. Future work will focus on more lightweight architectures, contrast-enhancement strategies, and more efficient and stable training schemes, as well as broader integration with downstream multi-modal tasks.

## Figures and Tables

**Table 1:** Quantitative comparison on the TNO dataset. The best results are highlighted in bold, and the second-best results are indicated with underlining. The symbol “↑” indicates that a higher value is better.

Dataset: TNO Infrared–Visible Fusion Dataset											
Methods	EN ↑	MI ↑	SF ↑	SCD ↑	VIF ↑	Q^IR/F^	Q^VI/F^	DBS	SSIM^IR^	SSIM^VI^	GBS
DenseFuse [15]	6.819	2.301	8.981	1.724	0.661	0.371	0.421	0.936	0.921	0.460	0.666
FusionGAN [31]	6.558	2.833	6.277	1.387	0.422	0.221	0.258	0.923	0.750	0.266	0.523
U2Fusion [41]	7.001	3.013	11.87	1.688	0.628	0.377	0.422	0.944	**0.929**	0.406	0.608
PIAFusion [29]	6.811	2.357	9.622	1.609	0.744	0.315	0.685	0.631	0.875	0.294	0.503
SeAFusion [47]	7.132	2.849	12.26	1.732	0.701	0.428	0.465	0.956	0.892	0.461	0.682
MUFusion [38]	7.106	3.448	10.04	1.567	0.540	0.331	0.365	0.950	0.843	0.353	0.590
IRFS [39]	6.617	3.137	8.707	**1.748**	0.591	0.305	0.460	0.798	0.909	0.406	0.618
IJMLC [34]	7.063	3.066	11.12	1.464	0.724	0.250	**0.784**	0.484	0.825	0.171	0.344
LIVFusion [37]	7.116	3.127	11.52	1.663	0.812	0.366	0.563	0.788	0.225	0.225	0.423
MBFTFuse (Ours)	**7.224**	**3.684**	**12.52**	1.747	**0.819**	**0.485**	0.525	**0.960**	0.904	**0.488**	**0.701**

**Table 2:** Quantitative comparison on the MSRS dataset. The best results are highlighted in bold, and the second-best results are indicated with underlining. The symbol “↑” indicates that a higher value is better.

Dataset: MSRS Infrared–Visible Fusion Dataset											
Methods	EN ↑	MI ↑	SF ↑	SCD ↑	VIF ↑	Q^IR/F^	Q^VI/F^	DBS	SSIM^IR^	SSIM^VI^	GBS
DenseFuse [15]	6.276	2.495	6.034	1.448	0.549	0.192	0.306	0.771	0.905	0.234	0.411
FusionGAN [31]	5.423	1.862	4.605	1.016	0.419	0.184	0.115	0.771	0.705	0.215	0.468
U2Fusion [41]	5.369	2.096	8.777	1.202	0.513	0.274	0.435	**0.772**	0.879	0.324	**0.538**
PIAFusion [29]	6.765	4.121	13.19	1.746	**0.992**	0.331	0.799	0.585	0.968	0.291	0.462
SeAFusion [47]	**6.797**	4.107	12.47	1.759	0.951	0.393	0.774	0.673	**0.974**	0.318	0.492
MUFusion [38]	6.196	1.670	10.46	1.360	0.581	0.286	0.472	0.755	0.900	0.317	0.521
IRFS [39]	6.664	2.218	10.57	1.764	0.697	0.265	0.566	0.638	0.926	**0.340**	0.537
IJMLC [34]	6.632	5.589	12.25	1.353	0.976	0.102	**0.932**	0.198	0.910	0.204	0.367
LIVFusion [37]	6.520	5.070	11.33	1.542	0.883	0.175	0.426	0.581	0.780	0.102	0.231
MBFTFuse (Ours)	6.780	**5.963**	**13.53**	**1.765**	0.978	**0.414**	0.773	0.698	0.949	0.328	0.513

**Table 3:** Quantitative comparison on the RoadScene dataset. The best results are highlighted in bold, and the second-best results are indicated with underlining. The symbol “↑” indicates that a higher value is better.

Dataset: RoadScene Infrared–Visible Fusion Dataset											
Methods	EN ↑	MI ↑	SF ↑	SCD ↑	VIF ↑	Q^IR/F^	Q^VI/F^	DBS	SSIM^IR^	SSIM^VI^	GBS
DenseFuse [15]	7.215	2.651	8.356	1.587	0.480	0.317	0.302	0.977	0.890	0.469	0.690
FusionGAN [31]	6.985	2.651	8.508	1.002	0.368	0.202	0.364	0.715	0.767	0.303	0.566
U2Fusion [41]	6.939	2.581	11.925	1.214	0.552	**0.585**	0.361	0.764	0.740	0.429	0.734
PIAFusion [29]	7.038	3.478	11.737	1.441	0.682	0.299	0.650	0.630	0.787	0.332	0.593
SeAFusion [47]	7.485	2.999	12.154	1.664	0.634	0.548	0.424	0.872	0.883	**0.633**	**0.835**
MUFusion [38]	7.435	2.101	12.898	1.428	0.503	0.356	0.401	0.941	0.847	0.451	0.695
IRFS [39]	7.046	2.721	9.793	1.468	0.573	0.434	0.425	**0.989**	0.695	0.205	0.456
IJMLC [34]	7.106	3.687	11.961	1.312	0.763	0.335	**0.689**	0.654	0.735	0.258	0.520
LIVFusion [37]	7.392	2.928	**15.866**	1.654	0.636	0.413	0.496	0.908	0.882	0.584	0.797
MBFTFuse (Ours)	**7.501**	**3.695**	13.438	**1.773**	**0.766**	0.406	0.578	0.824	**0.891**	0.611	0.814

**Table 4:** Quantitative comparison of network structure and module design on the TNO dataset. The best results are highlighted in bold, and the second-best results are indicated with underlining. The symbol “↑” indicates that a higher value is better.

Dataset: TNO Infrared–Visible Fusion Dataset											
Methods	EN ↑	MI ↑	SF ↑	SCD ↑	VIF ↑	Q^IR/F^	Q^VI/F^	DBS	SSIM^IR^	SSIM^VI^	GBS
w/o Conc	6.321	3.102	10.33	1.711	0.732	0.475	0.382	0.891	0.781	0.411	0.690
w/o VIS	6.012	2.968	7.351	1.682	0.642	0.421	0.287	0.811	0.859	0.362	0.593
w/o IR	5.926	3.207	10.65	1.703	0.612	0.305	0.422	0.839	0.802	0.452	**0.721**
w/o MCMB	6.863	3.461	10.13	1.684	0.786	0.507	0.395	0.876	0.874	0.431	0.661
w/o FTA	6.933	3.210	9.869	1.537	0.718	0.417	0.468	0.942	0.854	0.413	0.652
CBAM	7.124	3.412	12.13	**1.751**	0.793	0.467	**0.536**	0.931	0.889	0.451	0.673
GAM	7.210	3.607	11.76	1.732	0.803	0.477	0.519	0.958	**0.906**	0.475	0.688
Ours	**7.224**	**3.684**	**12.52**	1.747	**0.819**	**0.485**	0.525	**0.960**	0.904	**0.488**	0.701

**Table 5:** Quantitative comparison of network structure and module design on the MSRS dataset and RoadScene dataset. The best results are highlighted in bold, and the second-best results are indicated with underlining. The symbol “↑” indicates that a higher value is better.

Dataset	Methods	EN ↑	MI ↑	SF ↑	SCD ↑	VIF ↑	Q^IR/F^	Q^VI/F^	DBS	SSIM^IR^	SSIM^VI^	GBS
MSRS	w/o Conc	5.341	5.133	10.12	1.512	0.716	0.374	0.583	**0.782**	0.813	0.271	0.500
	w/o VIS	6.222	4.923	11.53	1.682	0.873	0.361	0.623	0.734	**0.959**	0.262	0.429
	w/o IR	5.754	4.237	12.03	1.641	0.811	0.316	0.703	0.620	0.822	**0.362**	**0.611**
	w/o MCMB	6.654	5.621	12.73	1.687	0.916	0.403	0.716	0.720	0.914	0.311	0.508
	w/o FTA	6.321	5.752	11.89	1.537	0.808	0.397	0.698	0.725	0.877	0.299	0.509
	Ours	**6.780**	**5.963**	**13.53**	**1.765**	**0.978**	**0.414**	**0.773**	0.698	0.949	0.328	0.514
Road-Scene	w/o Conc	6.876	3.007	11.23	1.301	0.683	0.371	0.507	0.845	0.821	0.556	0.808
	w/o VIS	7.113	3.318	12.32	**1.781**	0.712	**0.411**	0.476	**0.927**	0.871	0.514	0.742
	w/o IR	6.917	3.143	12.47	1.613	0.742	0.378	**0.592**	0.779	0.813	0.553	0.810
	w/o MCMB	7.361	3.476	13.23	1.745	0.776	0.379	0.585	0.786	0.861	0.567	0.794
	w/o FTA	7.127	3.472	12.71	1.619	0.703	0.387	0.518	0.855	0.843	0.588	**0.822**
	Ours	**7.501**	**3.695**	**13.44**	1.773	**0.766**	0.406	0.578	0.825	**0.891**	**0.611**	0.814

**Table 6:** Quantitative comparison of the dual-discriminator structure on the TNO dataset. The best results are highlighted in bold, and the second-best results are indicated with underlining. The symbol “↑” indicates that a higher value is better.

Methods	EN ↑	MI ↑	SF ↑	SCD ↑	VIF ↑	Q^IR/F^	Q^VI/F^	DBS	SSIM^IR^	SSIM^VI^	GBS
w/o *D_vis_*	5.163	2.936	8.255	1.612	0.785	0.421	0.361	0.850	0.882	0.453	0.679
w/o *D_ir_*	4.367	3.147	9.321	1.586	0.763	0.377	0.462	0.899	0.843	0.465	**0.711**
Ours	**7.224**	**3.684**	**12.52**	**1.747**	**0.819**	**0.485**	**0.525**	**0.960**	**0.904**	**0.488**	0.701

**Table 7:** Quantitative comparison of the loss function design on the TNO dataset. The best results are highlighted in bold, and the second-best results are indicated with underlining. The symbol “↑” indicates that a higher value is better.

Methods	*L_global_*	*L_pix_*	*L_adv_*	EN ↑	MI ↑	SF ↑	SCD ↑	VIF ↑	Q^IR/F^	Q^VI/F^	DBS	SSIM^IR^	SSIM^VI^	GBS
Case 1	√			6.952	2.875	10.62	1.721	0.806	0.433	0.379	0.933	0.876	0.433	0.662
Case 2	√	√		7.106	3.153	11.24	1.682	**0.821**	0.477	**0.528**	0.949	0.891	0.466	0.687
Case 3	√		√	7.036	3.523	10.33	1.713	0.802	0.458	0.511	0.945	0.877	0.471	0.699
Ours	√	√	√	**7.224**	**3.684**	**12.52**	**1.747**	0.819	**0.485**	0.525	**0.960**	**0.904**	**0.488**	**0.701**

**Table 8:** Parameter sensitivity analysis of $\mu_{2}$ on the TNO dataset. The best results are highlighted in bold, and the second-best results are indicated with underlining. The symbol “↑” indicates that a higher value is better.

$\mu_{2}$	EN ↑	MI ↑	SF ↑	SCD ↑	VIF ↑	Q^IR/F^	Q^VI/F^	DBS	SSIM^IR^	SSIM^VI^	GBS
0.5	6.152	2.334	9.62	**1.822**	0.746	0.428	0.379	0.939	0.868	0.413	0.645
0.8	**7.224**	**3.684**	**12.52**	1.747	**0.819**	**0.485**	0.525	**0.960**	0.904	**0.488**	**0.701**
1.0	7.124	3.334	11.97	1.643	0.802	0.471	**0.572**	0.903	0.884	0.463	0.687
1.2	6.224	3.450	9.22	1.776	0.783	0.423	0.495	0.922	**0.914**	0.421	0.631

**Table 9:** Quantitative comparison of the $L_{pix}$ design under different lighting conditions on the MSRS dataset. The best results are highlighted in bold. The symbol “↑” indicates that a higher value is better.

Scene	*L_global_*	*L_pix_*	*L_adv_*	EN ↑	MI ↑	SF ↑	SCD ↑	VIF ↑	Q^IR/F^	Q^VI/F^	DBS	SSIM^IR^	SSIM^VI^	GBS
DT1	√		√	6.653	5.671	**14.48**	1.742	**0.958**	0.271	**0.835**	0.490	0.933	**0.143**	**0.266**
DT2	√	√	√	**6.792**	**5.981**	14.41	**1.754**	0.955	**0.288**	0.809	**0.525**	**0.943**	0.140	0.258
NT1	√		√	5.421	3.645	8.31	1.824	0.972	0.632	0.648	**0.988**	0.938	0.509	0.509
NT2	√	√	√	**5.738**	**3.855**	**8.67**	**1.871**	**0.984**	**0.643**	**0.702**	0.956	**0.963**	**0.537**	**0.537**

**Table 10:** Performance comparison of object detection task on the MSRS dataset. The best results are highlighted in bold, and the second-best results are indicated with underlining.

Method	Precision			Recall			mAP [0.5:0.95]		
	Avg	Person	Car	Avg	Person	Car	Avg	Person	car
VIS	0.921	0.951	0.891	0.697	0.569	0.825	0.541	0.352	0.730
IR	0.888	0.893	0.883	0.734	**0.917**	0.551	0.619	**0.703**	0.534
DenseFuse [15]	0.862	0.918	0.806	0.663	0.620	0.706	0.576	0.590	0.562
FusionGAN [31]	0.854	0.887	0.820	0.654	0.682	0.625	0.558	0.536	0.580
U2Fusion [41]	0.880	0.923	0.836	0.790	0.765	0.815	0.658	0.603	0.712
PIAFusion [29]	0.888	0.942	0.833	0.838	0.812	0.863	0.675	0.624	0.725
SeAFusion [47]	0.907	0.955	0.858	0.817	0.821	0.812	0.707	0.665	0.748
MUFusion [38]	0.867	0.928	0.806	0.801	0.805	0.796	0.680	0.629	0.731
IRFS [39]	0.907	0.966	0.847	0.811	0.785	0.837	0.675	0.602	0.748
IJMLC [34]	0.900	0.924	0.876	0.758	0.646	**0.869**	0.554	0.386	0.722
LIVFusion [37]	0.931	**0.960**	0.901	**0.860**	0.883	0.837	0.706	0.662	**0.749**
MBFTFuse (Ours)	**0.932**	0.949	**0.914**	0.855	0.856	0.853	**0.708**	0.676	0.739

**Table 11:** Complexity comparison of different fusion methods under 640 × 480 input. The best results are highlighted in bold, and the second-best results are indicated with underlining.

	DenseFuse [15]	FusionGAN [31]	U2Fusion [41]	PIAFusion [29]	SeAFusion [47]
Params (M)	**0.088**	0.926	0.659	0.089	0.167
FLOPs (G)	**27.03**	284.50	202.36	27.34	50.99
	MUFusion [38]	IRFS [39]	IJMLC [34]	LIVFusion [37]	MBFTFuse (Ours)
Params (M)	0.463	0.242	2.132	0.456	1.471
FLOPs (G)	142.5	74.5	652.4	140.2	450.12

## Data Availability

To assist more researchers in this field, our code will be publicly available on https://github.com/esportsxi/MBFTFuse (accessed on 5 February 2026). The data presented in this study are available in the public domain: [TNO dataset: https://figshare.com/articles/dataset/TNO_Image_Fusion_dataset/1008029/2, MSRS dataset: https://github.com/Linfeng-Tang/MSRS, RoadScene Dataset: https://github.com/hanna-xu/RoadScene], last accessed: 5 February 2026.
